# Converging Paths: Artificial Intelligence for Integrating Biological and Digital Biomarkers in Personalized Longevity Medicine

**DOI:** 10.3390/ijms27219458

**Published:** 2026-10-23

**Authors:** Timur Saliev, Prim B. Singh

**Affiliations:** 1 Institute for Fundamental and Applied Medical Research, S.D. Asfendiyarov Kazakh National Medical University, Tole Bi Street 94, Almaty 050000, Kazakhstan; 2 Department of Biomedical Sciences, School of Medicine, Nazarbayev University, Astana 010000, Kazakhstan

**Keywords:** biological aging, artificial intelligence, aging clocks, digital biomarkers, multi-omics, wearable devices, proteomics, epigenetics, multimodal AI, precision medicine

## Abstract

Ageing is a multidimensional biological process characterised by interacting molecular, physiological, functional, and environmental changes that chronological age alone cannot adequately represent. The rapid development of epigenetic clocks, proteomic and metabolomic signatures, imaging-derived biomarkers, wearable sensors, and digital phenotypes has created an increasingly rich but fragmented landscape of aging biomarkers. Artificial intelligence (AI) offers a potential integrative framework that can combine these heterogeneous data streams and convert them into multidimensional estimates of biological age, health risk, functional reserve, and potentially treatment response. Recent studies demonstrate that molecular and clinical information can be integrated into composite aging measures, while independent work shows that wearable movement data, retinal imaging, and speech characteristics can provide non-invasive digital representations of aging-related phenotypes. The emerging concept of multimodal aging clocks extends these approaches by integrating physiological, clinical, imaging, and molecular measurements into a common representation of biological aging.

## 1. Introduction

Chronological age remains one of the strongest predictors of morbidity and mortality, but individuals of the same chronological age may differ substantially in biological aging rate, functional capacity, disease burden, and life expectancy [1,2]. This discrepancy has stimulated the development of biomarkers intended to quantify biological aging and to provide measurable intermediate outcomes for geroscience and longevity research [1,3]. The foundational human epigenetic clocks were developed by Horvath S. et al. [4], who demonstrated a multi-tissue DNA methylation clock using 353 CpG sites, and by Hannum et al. [5], who developed a blood-based methylation age predictor. These studies established that DNA methylation can estimate chronological age and reveal inter-individual differences in biological aging rate. They remain the reference points for subsequent epigenetic clocks such as PhenoAge and GrimAge.

However, molecular clocks capture only one layer of a multidimensional process. Digital biomarkers derived from wearables, smartphones, retinal imaging, and speech, together with imaging-derived phenotypes, provide complementary, repeatable, and functionally relevant information that cannot be inferred from a single molecular assay. The central rationale of this Perspective is therefore that AI may integrate these heterogeneous biological, digital, imaging, and clinical layers into multidimensional aging profiles that provide information beyond any individual aging clock. Such integration is not intended to create another single composite score, but to characterize which biological and functional systems are aging faster or slower in a given individual.

Subsequent models, including DNAm PhenoAge, demonstrated that aging-related molecular signatures could be associated not only with chronological age but also with mortality, disease, physical functioning, and other health outcomes [1].

Proteomic aging clocks have subsequently provided complementary information, with large-scale plasma protein measurements demonstrating associations between proteomic aging and multiple chronic diseases, multimorbidity, and mortality [6]. Despite these advances, most aging biomarkers remain modality-specific, meaning that each captures only a particular component of the aging process [1,6]. An epigenetic clock primarily reflects molecular regulation, whereas a proteomic clock may capture systemic signaling and organ-level processes, and wearable-derived measures may reflect physical activity, mobility, circadian behavior, or functional reserve [1,6,7].

Consequently, disagreement between different aging clocks should not necessarily be interpreted as measurement failure, because it may reflect genuine biological heterogeneity across tissues and physiological systems [8].

Several limitations of current aging clocks should be acknowledged. Most clocks are tissue- and platform-dependent, have been developed and validated predominantly in specific ancestries and healthcare systems, and may not generalize across populations. External validation is often limited, and many clocks have not been tested prospectively. In addition, molecular age and functional age are not equivalent: an epigenetic clock estimates molecular aging, whereas gait speed, cognitive performance, or frailty reflect functional consequences that may be influenced by disease, injury, or environment. Similarly, estimating biological age is not the same as predicting clinically relevant outcomes such as disease risk, functional decline, mortality, or treatment response. Multimodal approaches may address heterogeneity not by collapsing all measurements into a single score, but by preserving domain-specific information and explicitly modeling discordance between molecular, functional, and clinical systems.

## 2. From Isolated Aging Clocks to Multimodal Biological Age

The conceptual transition from individual biomarkers to integrated aging models is supported by increasingly large datasets in which multiple biological layers are measured within the same individuals [9]. A particularly important recent example is OMICmAge, which integrates proteomic, metabolomic, and clinical information with DNA-methylation-based measures and was developed using approximately 31,000 participants, with validation in independent cohorts [10]. The study illustrates the potential advantage of combining molecular and clinical dimensions, but it also shows that incremental predictive value over established single-modality clocks must be demonstrated rather than assumed. Reported gains are often modest, external validation across ancestries and healthcare systems remains limited, and clinical utility has not yet been established.

However, it remains uncertain whether multimodal integration consistently improves prediction beyond the best single-modality clock. Demonstrating incremental value requires pre-specified comparisons of calibration, discrimination, net reclassification improvement, integrated discrimination improvement, and decision-curve analysis. Combining modalities can improve performance when they capture complementary biology, but it can also add noise, increase overfitting, and reduce interpretability. Multimodal models should therefore be judged not by the number of data layers included, but by whether they provide reproducible incremental value for clinically meaningful outcomes.

A related development is the emergence of multimodal aging clocks that explicitly combine physiological, clinical, and molecular information [8]. In a multicentric Chinese cohort of more than 2000 individuals, Li and colleagues developed a hierarchical framework incorporating clinical physiological measurements and molecular information to characterize individual aging trajectories [8] (Table 1). Table 1 provides a conceptual map rather than a systematic appraisal. Scalability, clinical applicability, and predictive value are potential and context-dependent. Representative references: epigenetic; proteomic; metabolomic; digital/wearable; imaging; speech/cognitive and multimodal composite.

These studies support a shift from the question “How old is this person biologically?” toward the more clinically meaningful question “Which biological systems are aging faster, and how does this pattern relate to future functional and disease risk?” [11]. Such a multidimensional interpretation is particularly relevant because aging is increasingly understood as an interconnected network of molecular, cellular, organ-level, and behavioral processes rather than a single linear trajectory [12].

### Methodological Frictions in Multimodal Fusion

Although OMICmAge [10] and Li et al. [8] demonstrate feasibility, they also expose unresolved mathematical problems. The core difficulty is not simply data heterogeneity but structural asymmetry between modalities. Molecular measurements are typically sparse, irregular, and expensive, while wearable and smartphone data are dense, continuous, and noisy. Aligning an annual DNA methylation measurement with minute-level accelerometry, sleep, or heart-rate data requires explicit temporal aggregation, interpolation, and causal assumptions about the window of biological effect. Simple imputation can bias downstream age estimates because missingness is often informative: sicker individuals may have more frequent measurements, while healthier individuals may drop out. Multimodal models must therefore distinguish between missing-at-random, missing-not-at-random, and structurally unobserved modalities. Furthermore, high-frequency digital data can dominate the loss function, causing the model to underweight sparse molecular anchors unless architectures explicitly balance modality contributions. Finally, fusion can amplify batch effects: a platform-specific proteomic shift may be incorrectly coupled with a device-specific gait pattern, producing a spurious composite aging signal. These issues require probabilistic graphical models, multi-view learning with modality dropout, and explicit uncertainty propagation, rather than concatenation of features. OMICmAge and Li et al. are important but not yet definitive: they are largely observational, may not establish causality, and their composite scores have not been shown to change clinical management.

A further conceptual distinction is important. A multimodal biological-age score collapses heterogeneous information into a single number and may obscure clinically relevant discordance between organ systems. A multidimensional aging profile preserves domain-specific estimates—molecular, metabolic, cardiovascular, neurological, musculoskeletal, and functional—and can therefore identify which systems are aging faster or slower. Data harmonisation, missing-data handling, measurement timing, and platform variability are not secondary technical details; they determine whether a profile is reproducible and interpretable. Combining sparse molecular measurements with continuous digital data requires explicit temporal alignment, and different modalities may need different weighting depending on the clinical question.

## 3. Digital Biomarkers as a Dynamic Layer of Aging Assessment

Digital biomarkers provide a fundamentally different type of information because they can be collected repeatedly and, in some cases, continuously outside conventional clinical environments [7].

Wearable accelerometry, for example, can provide high-frequency measurements of movement, activity patterns, sedentary behavior, and physical function that are difficult to obtain through periodic clinical assessments [13].

McIntyre and colleagues demonstrated that machine-learning models could predict biological age from wearable-derived movement data and subsequently use predicted biological age to identify associations with nutritional and pharmacological interventions relevant to healthy aging [7]. Digital biomarkers can also capture aging phenotypes that are difficult to quantify molecularly [14,15].

Deep-learning analysis of retinal photographs has been shown to estimate biological age and to stratify morbidity and mortality risk, suggesting that the retina may function as a non-invasive window into systemic aging [14]. Similarly, automated analysis of spontaneous speech has recently demonstrated potential for estimating cognitive-domain performance and identifying individuals below normative cognitive thresholds in older adults [15,16].

These findings indicate that digital phenotyping could complement molecular aging clocks by providing longitudinal information about the functional consequences of biological aging. The critical opportunity is therefore not to replace molecular biomarkers with digital biomarkers but to connect them [7,14].

For example, changes in physical activity or gait could potentially be interpreted together with epigenetic age acceleration, proteomic age, inflammatory biomarkers, and metabolic profiles to distinguish transient functional changes from broader biological deterioration [17,18].

Despite this promise, the evidence that digital biomarkers specifically measure biological aging remains limited. Wearable, retinal, and speech signals can reflect functional aging, disease activity, medication effects, injury, mood, environment, or behavior rather than intrinsic aging. Device and platform differences, sampling frequency, wear time, language, culture, education, and recording conditions introduce substantial variability. Comorbidities and medications can alter gait, sleep, heart-rate variability, retinal features, and speech independently of aging. Therefore, digital biomarkers should currently be interpreted as functional and behavioral correlates of aging that may complement molecular clocks, not as direct measures of biological aging. Longitudinal and external validation is needed to determine whether changes in these signals predict meaningful outcomes beyond conventional clinical and molecular measures.

However, digital biomarkers are highly susceptible to confounding that can mimic biological aging. Acute illness, injury, surgery, depression, medication changes, seasonal variation, air quality, neighborhood walkability, occupational demands, and socioeconomic status can all alter gait, activity, sleep, heart-rate variability, speech, or retinal features. A model trained to predict biological age may therefore learn lifestyle noise or access-to-care differences rather than intrinsic aging. For example, a decrease in step count may reflect a knee injury, not accelerated musculoskeletal aging; reduced speech fluency may reflect language, education, or recording conditions rather than cognitive decline. These confounders are not merely technical: they can encode structural inequities and lead to biased predictions. Mitigation requires collection of contextual metadata, negative-control outcomes, causal diagrams, within-person designs, sensor-agnostic preprocessing, and sensitivity analyses that test whether associations persist after adjustment for acute events and socioeconomic factors. AI models should also be evaluated for whether they predict biological aging or merely proxy for health behaviors and healthcare access.

Longitudinal and external validation of digital biomarkers remains limited. Few studies have demonstrated that changes in wearable, retinal, or speech features predict mortality, functional decline, or treatment response independently of established clinical and molecular measures. Synchronising continuous digital data with intermittent molecular and clinical measurements is also challenging: annual blood draws and epigenetic screens cannot capture daily fluctuations, while continuous sensor data may contain acute perturbations that are not relevant to long-term aging. Practical solutions include pre-specified temporal windows, aggregation of digital data into clinically meaningful epochs, joint modeling of sparse and dense modalities, and external validation across devices, populations, and healthcare systems.

## 4. Artificial Intelligence as the Integrative Layer

The principal advantage of AI in this context is its capacity to model nonlinear relationships among high-dimensional variables that cannot easily be represented using conventional statistical approaches [11].

Deep-learning architectures can identify latent representations across molecular and clinical datasets, while multimodal architectures can potentially learn relationships between structured laboratory data and unstructured imaging, speech, or sensor-derived information [19,20].

Recent generative AI approaches further demonstrate the possibility of reconstructing relationships between partially observed molecular modalities and integrating heterogeneous biological datasets into unified representations [21]. The emergence of multimodal foundation models may therefore represent an important next step in longevity biomarker research.

However, a clear distinction should be maintained between established machine-learning applications and emerging generative or foundation-model approaches. Conventional supervised models have been used for age prediction and risk stratification, but multimodal foundation models remain largely experimental. They require large, diverse, and well-curated datasets; they are vulnerable to data leakage, overfitting, batch effects, and model drift; and they have not been prospectively validated in clinical settings. AI-generated aging profiles would need external validation, calibration, and demonstration of incremental clinical value beyond existing biomarkers and risk models. Until then, they should be regarded as research tools rather than clinically ready instruments.

From a geroscience perspective, the central challenge for AI is not simply to fuse modalities but to respect the hierarchical, asynchronous, and organ-specific nature of aging biology. Molecular, functional, and digital layers evolve on different timescales: epigenetic and proteomic changes may unfold over months to years, whereas gait, sleep, and speech can fluctuate within days. AI models that ignore these temporal hierarchies risk conflating acute perturbations with intrinsic aging. Because ageing is heterogeneous across physiological systems, a clinically useful AI system should preserve discordance among molecular, metabolic, cardiovascular, neurological, musculoskeletal, and functional domains rather than collapse them into a single composite score. We therefore argue that multimodal AI should be evaluated as a tool for generating domain-specific risk profiles and mechanistic hypotheses, not as a black-box longevity estimator. Its value will depend on whether it can distinguish genuine biological heterogeneity from technical noise, lifestyle confounding, and socioeconomic bias. This distinction requires causal inference and external validation, not only predictive accuracy.

Instead of developing independent models for epigenetic age, proteomic age, gait, retinal age, and cognitive function, a multimodal architecture could theoretically generate a shared representation of biological aging from multiple complementary inputs [22].

The output would ideally not be a single opaque “longevity score” but a structured biological-aging profile describing molecular, metabolic, cardiovascular, neurological, musculoskeletal, and functional dimensions separately [23]. This distinction is clinically important because a single composite score could conceal biologically meaningful discordance. An individual might have relatively favorable epigenetic age but accelerated cardiovascular or functional aging, while another person could demonstrate molecular age acceleration without measurable functional decline [24,25].

Table 2 should be interpreted as a conceptual map rather than a systematic evidence appraisal. Rows describing multi-omics integration, molecular-clinical integration, and imaging-molecular integration are supported by observational studies, but prospective clinical validation remains limited. Rows describing multimodal foundation models, clinical decision support, and closed-loop longevity monitoring are largely speculative. Important technical risks include interpretability, bias, data leakage, overfitting, external validation, and model drift. Explainability methods can identify which features contributed to a prediction, but they do not establish biological causality. Clinical decision support and closed-loop monitoring should therefore be qualified as research objectives requiring prospective randomized or pragmatic intervention studies before they can be considered established clinical applications.

## 5. Toward Dynamic and Personalized Longevity Medicine

The ultimate value of multimodal aging biomarkers will depend on whether they can improve clinical decisions rather than merely predict chronological or biological age. A clinically useful system should ideally identify modifiable risk, predict clinically meaningful outcomes, monitor longitudinal changes, and determine whether an intervention produces a favorable biological response [26]. This would enable a transition from one-time assessment toward dynamic monitoring of biological trajectories.

In this context, clinically meaningful outcomes should be explicitly defined and may include mortality, incident multimorbidity, functional decline, frailty, cognitive impairment, quality of life, and treatment response. Changes in a biological-age score are not automatically equivalent to changes in these outcomes. Repeated multimodal data collection also faces practical challenges, including cost, patient burden, adherence, missing data, measurement variability, device differences, and mismatched sampling frequencies. These factors may limit real-world feasibility and must be incorporated into study design and health-economic evaluation.

For example, a personalized longevity platform could combine baseline clinical information with DNA methylation, plasma proteomics, metabolomics, retinal imaging, wearable activity, sleep characteristics, and cognitive or speech assessments [27,28].

AI could then estimate multidimensional biological age and identify domains that are statistically associated with the individual’s risk profile [29,30]. Such associations should not be interpreted as causal, and predictive importance does not necessarily indicate that modifying a domain will change clinical outcomes.

For example, consider a 62-year-old man with obesity, hypertension, and mild cognitive complaints. At baseline, the system could integrate DNA methylation age, plasma proteomic and metabolomic signatures, retinal imaging, wearable-derived gait and sleep parameters, and speech-based cognitive features. The AI system would not output a single longevity score but rather a multidimensional profile indicating, for example, accelerated cardiovascular and metabolic aging but relatively preserved epigenetic age. After a 6-month lifestyle intervention, repeated measurements could show improvement in metabolic and functional domains but no change in epigenetic age. This would allow the clinician to distinguish a favorable functional response from a broader biological effect and to adjust the intervention accordingly.

Prospective and intervention-based validation is required to determine whether changes in multimodal aging profiles reliably reflect responses to lifestyle or pharmacological interventions. Until such evidence is available, these profiles should be regarded as hypothesis-generating and decision-support tools.

### Clinical Accountability and the Intervention-Response Gap

A critical unresolved question is what a clinician should do when a multimodal model reports accelerated cardiovascular aging relative to epigenetic age. At present, there is no validated intervention protocol triggered by discordance between organ-specific aging clocks. Longevity medicine lacks randomized controlled trial evidence that altering a composite AI-derived age score extends healthspan, reduces multimorbidity, or improves functional independence. Most existing clocks are prognostic: they predict mortality or disease risk, but they do not establish that the predicted risk is modifiable or that changing the score changes outcomes. Without validated intervention-response biomarkers, multimodal platforms risk becoming expensive prognostic mirrors rather than therapeutic tools. To move beyond this, the field needs target trial emulation, pragmatic randomized trials with aging-related functional endpoints, and pre-specified thresholds for clinical action. Composite scores should be accompanied by decision rules, uncertainty intervals, and evidence that specific interventions change specific aging domains. Until then, these systems should be framed as hypothesis-generating and decision-support tools, not as autonomous diagnostic or therapeutic instruments.

From a clinical translation perspective, the field should move from composite longevity scores toward domain-specific, action-linked aging profiles. A profile is clinically meaningful only if it maps onto validated interventions. For example, accelerated metabolic aging might prompt intensified lifestyle or glucose-lowering therapy, whereas accelerated musculoskeletal aging might trigger resistance training or fall-prevention programs. At present, such mapping is largely absent. Most multimodal clocks remain prognostic and observational; they predict risk but do not demonstrate that modifying a predicted domain alters morbidity, frailty, or mortality. We therefore propose that clinical translation should proceed through target trial emulation and pragmatic randomized trials with pre-specified, domain-specific endpoints. Until such evidence exists, AI-generated aging profiles should be presented as decision-support hypotheses with explicit uncertainty, and they should not imply therapeutic actionability. This caution is particularly important in longevity medicine, where commercial enthusiasm may outpace evidence.

## 6. The Major Barrier: Validation Rather than Algorithmic Performance

The principal challenge facing multimodal longevity AI is unlikely to be the ability to construct increasingly complex models, but rather the demonstration that these models provide reproducible and clinically meaningful information across populations [22,31].

Validation should be explicitly staged. Internal validation assesses reproducibility within the development dataset. External validation tests performance in independent datasets, institutions, or populations. Prospective validation evaluates whether the model predicts outcomes in data collected after model development. Each stage provides different levels of evidence. Class imbalance, missing data, and differential availability of modalities can substantially affect performance and must be reported. Calibration, decision-curve analysis, and clinically relevant performance measures should be prioritized alongside discrimination, because a model may rank patients well but still produce poorly calibrated or clinically unhelpful risk estimates.

A model trained predominantly in one ancestry, healthcare system, socioeconomic environment, or age distribution may perform substantially differently in another population [32]. This issue is especially important for aging research because environmental exposures, genetic background, disease prevalence, medication use, lifestyle, and access to healthcare vary substantially between populations [33,34].

Data heterogeneity represents another major limitation because laboratory assays, wearable devices, imaging protocols, sampling intervals, and preprocessing pipelines can introduce systematic differences that AI models may inadvertently learn as biological signals.

Temporal drift and confounding further complicate validation. Aging is dynamic, but so are measurement technologies, clinical coding practices, wearable firmware, and population health behaviors. A model trained on data from one era may learn device-specific artifacts or secular trends rather than biological aging. Temporal external validation, drift detection, continual recalibration, and time-varying covariate models are therefore essential. Confounding is equally challenging: socioeconomic status, environmental exposures, medication use, and access to care can influence both molecular and digital biomarkers, creating non-causal associations that AI may exploit. Causal inference methods, including directed acyclic graphs, negative controls, instrumental variables, and longitudinal within-person analyses, should complement predictive modeling. Without these safeguards, multimodal aging models may reproduce existing health disparities while appearing technically accurate.

Interpretability is also critical because clinicians may be reluctant to act on an unexplained composite aging score [35,36]. A clinically useful multimodal system should identify which measurements contributed to the prediction, quantify uncertainty, distinguish correlation from causation, and provide evidence linking predicted risk to clinically actionable outcomes [37].

However, explainability methods do not demonstrate biological causality. Feature importance, saliency maps, and attention weights indicate model behavior, not causal mechanisms. Causal claims require experimental or quasi-experimental designs, target trial emulation, or Mendelian randomization where appropriate.

### Practical Solutions for Data Standardization, Missing Data, and Validation

Several concrete steps can mitigate these problems. First, all data should be mapped to common semantic standards, including OMOP CDM or FHIR for clinical data, LOINC for laboratory measurements, SNOMED CT for diagnoses, and HPO or EFO for phenotype and molecular annotations. Second, wearable data should be resampled to common temporal epochs, such as 1-min, 5-min, or 1-h windows, and summarized using device-agnostic features including mean, standard deviation, entropy, circadian amplitude, and wear time. Third, missing data should be handled using multiple imputation, masked-loss neural networks, missingness indicators, and sensitivity analyses. Fourth, batch and platform effects should be corrected using ComBat, mixed-effects models, or domain-adaptation approaches. Fifth, imaging data should follow DICOM standards and radiomics quality criteria. Finally, validation should be prospective, external, temporal, and multi-ancestry, and should report calibration, discrimination, decision curves, and TRIPOD-AI or PROBAST-AI criteria (Table 3).

## 7. Federated Learning and the Future of Distributed Longevity Research

The integration of molecular and digital biomarkers creates a substantial privacy challenge because the resulting datasets may combine genomic information, health records, behavioral patterns, continuous sensor data, imaging, and potentially speech [38,39].

Federated learning offers a potential strategy for collaborative model development in which institutions can train shared models without transferring raw patient-level data to a central repository [40].

However, federated learning should not be regarded as automatically guaranteeing privacy because model updates themselves can potentially leak information and because distributed datasets may remain heterogeneous and biased.

In practice, federated learning also faces communication costs, computational requirements, differences in local infrastructure, and difficulties in harmonising datasets across institutions. Privacy preservation is not equivalent to data security: federated learning alone does not prevent information leakage. Secure aggregation, differential privacy, homomorphic encryption, and secure multiparty computation are therefore essential complements. Federated learning also does not guarantee representative or unbiased datasets, because participating institutions and their populations may still differ substantially.

Recent work emphasizes that successful clinical federated learning requires attention to privacy-preserving mechanisms, data heterogeneity, robustness, communication efficiency, governance, and real-world validation [41].

For longevity research, federated learning could nevertheless enable geographically distributed cohorts to contribute to common multimodal aging models while retaining sensitive molecular and behavioral information locally [41].

International collaboration should be linked explicitly to external validation and generalisability, so that multimodal aging models are tested across healthcare systems, ancestries, and data infrastructures rather than only within the institutions that developed them.

### Practical Federated Learning Solutions

Federated learning should be implemented with secure aggregation, differential privacy, homomorphic encryption, and secure multiparty computation. Algorithms such as FedAvg, FedProx, and personalized federated learning can help address non-IID data across hospitals and cohorts. Communication efficiency can be improved through quantization, sparsification, and asynchronous updates. Governance should include data-use agreements, model cards, audit trails, and equitable participation of underrepresented populations. These measures are essential for translating federated longevity models into real-world healthcare settings.

## 8. A Proposed Framework for Integrated Longevity Biomarkers

This framework is conceptual and is not yet supported by prospective empirical validation. It is intended to organize existing evidence and define a research agenda.

A practical architecture for personalized longevity medicine could therefore consist of four interconnected layers [8,11]. The first layer would comprise molecular biomarkers, including DNA methylation, transcriptomics, proteomics, metabolomics, inflammatory markers, and other circulating indicators [6]. The second layer would include digital biomarkers derived from wearables, smartphones, retinal imaging, speech, sleep, activity, and other continuously or repeatedly measurable phenotypes [7].

The third layer would incorporate clinical and functional information, including comorbidities, laboratory parameters, medication exposure, cognitive performance, gait, physical performance, and patient-reported outcomes [8].

**Figure 1:**
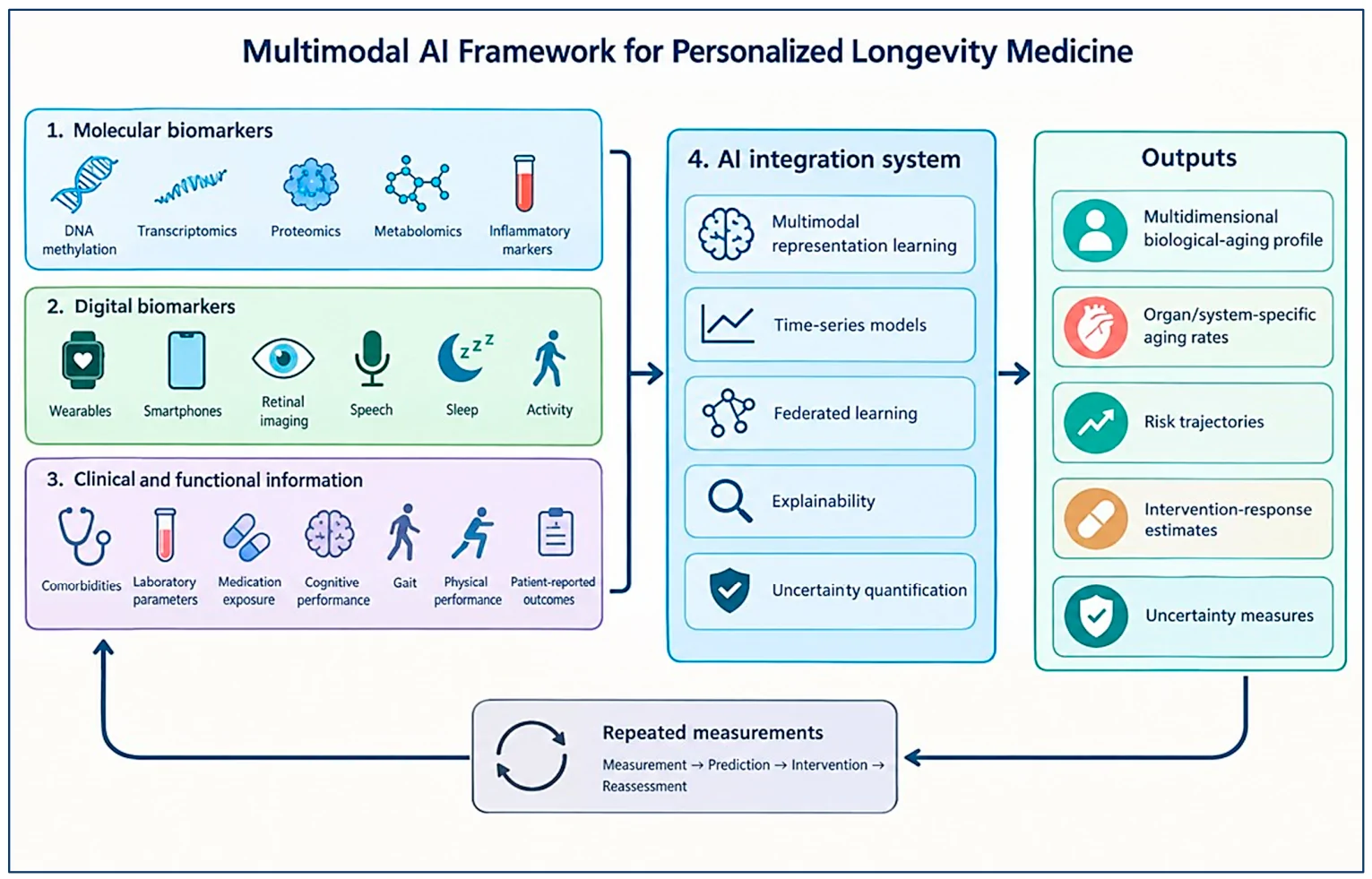
Multimodal AI framework for personalized longevity medicine. The figure was created by the authors; AI was used only for language editing and schematic refinement, not for image generation.

The four layers are not mutually exclusive. For example, gait can be captured by wearables or clinical assessment, and cognitive performance can be measured by speech or formal testing. The layers should therefore be understood as functional categories rather than rigid partitions. The fourth layer would be an AI integration system generating a multidimensional biological-aging profile, risk estimates, trajectory analysis, and uncertainty measures rather than a single unqualified longevity score [8] (Figure 1).

Uncertainty and confidence in individual predictions should be communicated explicitly, for example through prediction intervals, probabilistic outputs, and calibration plots. Data harmonisation, missing-data handling, measurement timing, and domain weighting are central to implementation and should be pre-specified.

The most important innovation would be the longitudinal integration of these layers [7]. First, retrospective harmonization of existing cohorts should define common data elements and minimum reporting standards. Second, prospective multimodal cohorts should collect synchronized molecular, digital, imaging, and clinical data. Third, federated learning should be used to train and validate models across institutions without transferring raw data. Fourth, pragmatic clinical trials should test whether multimodal aging profiles change clinical decisions or improve outcomes such as frailty, multimorbidity, functional decline, or mortality. Each stage should include pre-specified endpoints, external validation, calibration, and decision-curve analysis.

This approach could eventually establish a form of “closed-loop longevity medicine” in which measurement, prediction, intervention, and reassessment continuously inform one another, although this concept currently remains a research objective rather than an established clinical paradigm.

Demonstrating that closed-loop, biomarker-guided decisions improve clinically meaningful outcomes will require prospective intervention studies with pre-defined endpoints.

## 9. Conclusions

Longevity research is moving from isolated aging biomarkers toward integrated, multidimensional models of biological aging. AI may facilitate this transition by jointly interpreting heterogeneous molecular, clinical, imaging, wearable, and digital biomarkers over time. However, current systems remain research tools rather than established clinical instruments. Integrated AI systems may eventually enable earlier detection of biological deterioration, personalized prevention, intervention monitoring, and improved characterization of healthspan trajectories, but these remain potential applications requiring prospective validation. Major unresolved challenges include external validation, population diversity, data harmonisation, missing data, interpretability, privacy protection, and the definition of clinically meaningful endpoints. It is essential to distinguish biological-age estimation from demonstrated improvement in healthspan or clinical outcomes. Multimodal aging clocks currently estimate risk; they do not prove that modifying the estimated risk alters healthspan. The field should prioritize intervention-response validation, standardized endpoints, and evidence that composite aging profiles change clinical decisions or outcomes. The future of longevity medicine may depend on transparent AI frameworks that combine complementary biomarkers into individualized models of aging, but clinical actionability remains a key research objective rather than an established achievement.

## Figures and Tables

**Table 1:** Complementary biological and digital biomarkers for multidimensional assessment of aging.

Integration Level	AI Approach	Application in Longevity Medicine	Evidence Level	Main Limitation
Multi-omics	Deep learning; multimodal neural networks	Integration of epigenomic, proteomic, metabolomic, and transcriptomic data	Demonstrated (observational)	High dimensionality; batch effects; limited external validation
Molecular–clinical	Machine learning; composite aging models	Combining molecular clocks with laboratory values, comorbidities, and physiological parameters	Demonstrated (observational)	Clinical heterogeneity; no proven improvement in clinical outcomes
Imaging–molecular	Deep learning; computer vision	Linking retinal or other imaging features with systemic and molecular aging	Emerging	Imaging protocol variability; population bias; need for multi-centre validation
Digital–molecular	Multimodal ML; representation learning	Linking wearable-derived activity and physiology with molecular aging clocks	Emerging	Lack of synchronized datasets; confounding by lifestyle and acute illness
Speech–cognitive	Natural language processing; speech AI	Combining speech features with cognitive and neurological biomarkers	Emerging	Language, culture, education, and recording conditions
Functional–digital	Time-series ML; sensor-based AI	Combining gait, activity, sleep, and physiological signals	Emerging	Missing data; device-specific measurement differences
Multimodal foundation models	Transformers; multimodal representation learning	Joint analysis of clinical, molecular, imaging, and digital data	Speculative	Large data requirements; opacity; computational cost; model drift
Clinical decision support	Explainable AI; multimodal prediction	Individualized prevention and monitoring recommendations	Speculative	No evidence that AI recommendations improve patient outcomes
Closed-loop longevity monitoring	Continuous multimodal AI	Repeated measurement → prediction → intervention → reassessment	Speculative	No validated intervention protocols or clinically meaningful endpoints

**Table 2:** AI-enabled integration of aging biomarkers: current opportunities and translational challenges.

Integration Level	AI Approach	Potential Application in Longevity Medicine	Expected Clinical Value	Main Translational Challenge	Priority for Future Research
Multi-omics integration	Deep learning; multimodal neural networks	Integration of epigenomic, proteomic, metabolomic and transcriptomic data	More comprehensive characterization of biological aging	High dimensionality, batch effects and biological interpretability	External validation and identification of clinically actionable molecular signatures
Molecular–clinical integration	Machine learning; composite aging models	Combining molecular clocks with laboratory values, comorbidities and physiological parameters	Improved risk stratification and biological-age estimation	Clinical heterogeneity and temporal changes in biomarkers	Prospective validation against morbidity, mortality and functional outcomes
Digital–molecular bridging	Multimodal ML; representation learning	Linking wearable-derived activity/physiology with molecular aging clocks	Continuous monitoring of changes associated with biological aging	Lack of synchronized molecular and digital datasets	Longitudinal cohorts with repeated molecular and wearable measurements
Imaging–molecular integration	Deep learning; computer vision	Linking retinal or other imaging features with systemic/molecular aging	Non-invasive estimation of biological and organ-specific aging	Imaging protocol variability and population bias	Multicentre validation across ethnicities, devices and healthcare systems
Speech–cognitive integration	Natural-language processing; speech AI	Combining speech-derived features with cognitive and neurological biomarkers	Remote monitoring of cognitive aging	Language, culture, education and recording conditions	Multilingual and cross-cultural validation
Functional–digital integration	Time-series ML; sensor-based AI	Combining gait, activity, sleep and physiological signals	Early identification of functional decline	Missing data and device-specific measurement differences	Standardized digital phenotypes and clinically meaningful endpoints
Multimodal foundation models	Transformers; multimodal representation learning	Joint analysis of structured clinical data, molecular measurements, imaging and digital phenotypes	Potentially unified biological-aging representation	Large data requirements, opacity, computational cost and model drift	Explainable and clinically validated foundation models
Longitudinal trajectory modeling	Time-series AI; recurrent/transformer models	Tracking biological-age trajectories rather than single measurements	Detection of accelerated deterioration or response to intervention	Need for repeated standardized measurements	Prospective intervention studies with predefined trajectory endpoints
Federated learning	Distributed/federated AI	Collaborative training across hospitals and research centers without centralizing raw patient data	Larger and more diverse datasets while improving data governance	Privacy leakage, heterogeneous datasets, communication and governance issues	Multicentre prospective validation and privacy-preserving architectures
Clinical decision support	Explainable AI; multimodal prediction models	Individualized prevention and monitoring recommendations	Translation of biomarker information into clinical decisions	Lack of evidence that AI recommendations improve patient outcomes	Randomized or pragmatic clinical studies
Closed-loop longevity monitoring	Continuous multimodal AI	Repeated measurement → prediction → intervention → reassessment	Potential transition from static risk assessment to dynamic healthspan management	No established clinical standards or validated intervention-response biomarkers	Define clinically meaningful endpoints and demonstrate outcome benefit

**Table 3:** Practical solutions for data standardization, missing-data handling, and validation in multimodal longevity AI.

Challenge	Practical Solution	Standards/Tools	Validation/Reporting
Varying wearable sampling rates	Resample to common epochs; extract device-agnostic summary features; record wear time and sensor metadata	1-min/5-min/1-h windows; raw accelerometry where possible	Report preprocessing, wear-time algorithms, and device heterogeneity
Missing data	Multiple imputation; masked-loss neural networks; missingness indicators; sensitivity analyses	MICE, missForest, GAIN, SAITS, BRITS	Compare complete-case, imputed, and probabilistic models
Batch/platform effects	Statistical harmonization and domain adaptation	ComBat, limma, mixed-effects models, adversarial learning	External validation across platforms and sites
Semantic heterogeneity	Map to common data models and ontologies	OMOP CDM, FHIR, LOINC, SNOMED CT, HPO, EFO	Report mapping quality and unmapped codes
Imaging variability	Protocol harmonization; quality control; batch correction	DICOM, Radiomics Quality Score, ComBat	Multi-centre and multi-device validation
Temporal drift	Time-aware validation; drift detection; recalibration	Time-series ML, continual learning, monitoring dashboards	Prospective and temporal external validation
Confounding by acute illness, lifestyle, socioeconomic & environmental exposures	Collect contextual metadata; use negative controls, causal diagrams, within-person designs, and sensitivity analyses	DAGs, target trial emulation, mixed-effects models, E-values	Test whether associations persist after adjustment for acute events and social determinants
Privacy and governance	Privacy-preserving distributed training	Secure aggregation, differential privacy, homomorphic encryption, SMPC, FedProx	Model cards, audit trails, data-use agreements
Clinical usefulness	Pre-specified endpoints and decision-curve analysis	TRIPOD-AI, PROBAST-AI, CONSORT-AI, SPIRIT-AI	Mortality, multimorbidity, frailty, gait, cognition, intervention response

## Data Availability

No new data were created or analyzed in this study. Data sharing is not applicable to this article.

## References

[1] Levine M.E., Lu A.T., Quach A., Chen B.H., Assimes T.L., Bandinelli S., Hou L., Baccarelli A.A., Stewart J.D., Li Y. (2018). An epigenetic biomarker of aging for lifespan and healthspan. Aging.

[2] Hansen C.W., Strulik H. (2025). Biological age across the globe: 1990–2019. J. Econ. Ageing.

[3] Ho K.M., Morgan D.J., Johnstone M., Edibam C. (2023). Biological age is superior to chronological age in predicting hospital mortality of the critically ill. Intern. Emerg. Med..

[4] Horvath S. (2013). DNA methylation age of human tissues and cell types. Genome Biol..

[5] Hannum G., Guinney J., Zhao L., Zhang L., Hughes G., Sadda S., Klotzle B., Bibikova M., Fan J.-B., Gao Y. (2013). Genome-wide Methylation Profiles Reveal Quantitative Views of Human Aging Rates. Mol. Cell.

[6] Argentieri M.A., Xiao S., Bennett D., Winchester L., Nevado-Holgado A.J., Ghose U., Albukhari A., Yao P., Mazidi M., Lv J. (2024). Proteomic aging clock predicts mortality and risk of common age-related diseases in diverse populations. Nat. Med..

[7] McIntyre R.L., Rahman M., Vanapalli S.A., Houtkooper R.H., Janssens G.E. (2021). Biological Age Prediction From Wearable Device Movement Data Identifies Nutritional and Pharmacological Interventions for Healthy Aging. Front. Aging.

[8] Li J., Jiang B., Zhang W., Hao J., Liu Z., Ji Q., Zheng Y., Lu X., Zheng Z., Ma S. (2026). Multimodal clocks of human aging. Cell.

[9] Vasileiou M., Liposits G., Barakat B., Nguyen N.P. (2026). Multi-Omics Signatures of Organ Clocks in Biological Aging and Disease: A Conceptual Framework for Organ-Specific Aging Clocks. Aging Cell.

[10] Chen Q., Dwaraka V.B., Carreras-Gallo N., Armstrong J.F., Sehgal R., Argentieri M.A., Richmond A., Aparicio A., Mendez K., Chen Y. (2026). OMICmAge quantifies biological age by integrating multi-omics with electronic medical records. Nat. Aging.

[11] Chen J., Ren Y., Zhou Y., Wang Z., Mao K., Yu Z., Li J., Guo X., Xu H., Wang Y. (2026). A generative AI framework unifies human multi-omics to model aging, metabolic health, and intervention response. Cell Metab..

[12] López-Otín C., Blasco M.A., Partridge L., Serrano M., Kroemer G. (2023). Hallmarks of aging: An expanding universe. Cell.

[13] Macias Alonso A.K., Hirt J., Woelfle T., Janiaud P., Hemkens L.G. (2024). Definitions of digital biomarkers: A systematic mapping of the biomedical literature. BMJ Health Care Inform..

[14] Nusinovici S., Rim T.H., Yu M., Lee G., Tham Y.-C., Cheung N., Chong C.C.Y., Da Soh Z., Thakur S., Lee C.J. (2022). Retinal photograph-based deep learning predicts biological age, and stratifies morbidity and mortality risk. Age Ageing.

[15] Heitz J., Engler I.M., Langer N. (2026). Towards a speech-based digital biomarker for cognitive impairment: Speech as a proxy for cognitive assessment. npj Digit. Med..

[16] Tseng R.M.W.W., Rim T.H., Shantsila E., Yi J.K., Park S., Kim S.S., Lee C.J., Thakur S., Nusinovici S., Peng Q. (2023). Validation of a deep-learning-based retinal biomarker (Reti-CVD) in the prediction of cardiovascular disease: Data from UK Biobank. BMC Med..

[17] Ammous F., Peterson M.D., Mitchell C., Faul J.D. (2025). Physical Activity Is Associated with Decreased Epigenetic Aging: Findings from the Health and Retirement Study. J. Cachexia Sarcopenia Muscle.

[18] Shan J., Tay J.H., Ye K.X., Guo J., Cao L., Zeng Y., Lee T.-S., Heok K.E., Kennedy B.K., Maier A.B. (2026). Lifestyle factors and DNA methylation-based aging clocks: Cross-sectional and longitudinal associations in the Singapore diet and healthy aging cohort. J. Prev. Alzheimer’s Dis..

[19] Razzaq K., Shah M. (2025). Machine Learning and Deep Learning Paradigms: From Techniques to Practical Applications and Research Frontiers. Computers.

[20] Ros H., Chan N., Cook M.T., Shorthouse D. (2026). Artificial intelligence and machine learning guided optimization in drug delivery. Adv. Drug Deliv. Rev..

[21] Elhanashi A., Essahraui S., Dini P., Paolini D., Zheng Q., Saponara S. (2026). Generative AI and the Foundation Model Era: A Comprehensive Review. Big Data Cogn. Comput..

[22] Haugg F., Lee G., He J., Johnson J., Zapaishchykova A., Bitterman D.S., Kann B.H., Aerts H.J.W.L., Mak R.H. (2025). Imaging biomarkers of ageing: A review of artificial intelligence-based approaches for age estimation. Lancet Healthy Longev..

[23] Sehgal R., Markov Y., Qin C., Meer M., Hadley C., Shadyab A.H., Casanova R., Manson J.E., Bhatti P., Moore A.Z. (2025). Systems Age: A single blood methylation test to quantify aging heterogeneity across 11 physiological systems. Nat. Aging.

[24] Wu Y., Zhang T., Li S., Leung J., Kwok T., Magaziner J. (2026). Utility of biological aging markers for mortality risk stratification in community-dwelling older adults: Insights from the Mr. OS and Ms. OS (Hong Kong) cohort. J. Gerontol. Ser. A Biol. Sci. Med. Sci..

[25] Zhou S., Chen G., Fong T.-L., Tang G., Xiong R., Sun Y.X., Lu J., Wang N., Feng Y. (2025). Joint association of frailty index and biological aging with all-cause and cause-specific mortality: A population-based longitudinal cohort study. Arch. Gerontol. Geriatr..

[26] Ludwig C.A., Salvi A., Mesfin Y., Arnal L., Langlotz C., Mahajan V. (2026). A multimodal retinal aging clock for biological age prediction and systemic health assessment via OCT and fundus imaging. Sci. Rep..

[27] Furrer R., Handschin C. (2025). Biomarkers of aging: From molecules and surrogates to physiology and function. Physiol. Rev..

[28] Yusri K., Kumar S., Fong S., Gruber J., Sorrentino V. (2024). Towards Healthy Longevity: Comprehensive Insights from Molecular Targets and Biomarkers to Biological Clocks. Int. J. Mol. Sci..

[29] Qiu W., Chen H., Kaeberlein M., Lee S.-I. (2023). ExplaiNAble BioLogical Age (ENABL Age): An artificial intelligence framework for interpretable biological age. Lancet Healthy Longev..

[30] Wang G., Ding P., Li Z., Tang Q., Tu L., Jiang T., Zhang L., Su B., Xu J., An H. (2026). Artificial intelligence approaches in biological age prediction: Current status and challenges. Br. J. Biomed. Sci..

[31] Zhao S., Yu P., Xu R., Li S. (2026). The role of artificial intelligence in cancer epidemiology: Challenges and opportunities. Intell. Oncol..

[32] Pati S., Kumar S., Varma A., Edwards B., Lu C., Qu L., Wang J.J., Lakshminarayanan A., Wang S.-H., Sheller M.J. (2024). Privacy preservation for federated learning in health care. Patterns.

[33] Vereecke S., Bennett K., Schrempft S., Kobor M., Brauer M., Stringhini S. (2025). The Intersection of Socioeconomic and Environmental Factors in Aging: Insights from a Narrative Review. Int. J. Environ. Res. Public Health.

[34] Schaid D.J., McDonnell S.K., Akhtari F.S., Sinnwell J.P., Batzler A., Cobran E.K., Motsinger-Reif A. (2025). Polygenic scores and social determinants of health: Their correlations and potential biases. Hum. Genet. Genom. Adv..

[35] Yu G., Shao Z., Zhuo J., Chen Z. (2026). Immune Ageing Clocks: A Methods-Oriented Review of Tasks, Modalities, Models, and Recalibration. Cells.

[36] Li Y., Zhang X., Li X., Zhang Z. (2026). Brain maintenance biomarkers from structural and functional interactions in aging and neurodegeneration. Nat. Commun..

[37] Miteloudis A., Hatzilygeroudis I. (2026). An Overview of Recent Interpretability and Explainability Approaches for Tree-Based Ensembles. Information.

[38] Süwer S., Ullah M.S., Probul N., Maier A., Baumbach J. (2024). Privacy-by-Design with Federated Learning will drive future Rare Disease Research. J. Neuromuscul. Dis..

[39] Pratihar R., Sankar R. (2025). Integrative Federated Learning Framework for Multimodal Parkinson’s Disease Biomarker Fusion. Computers.

[40] Rahmani A.H., Sarwar T. (2026). Artificial Intelligence and Digital Biomarkers for Early Detection and Monitoring of Neurological Disorders: A Narrative Review. Diagnostics.

[41] Bakas S., Li X., Shah P., Roth H.R. (2026). Federated Learning in Healthcare: From Research to Real-World Deployment. Annu. Rev. Biomed. Eng..

